# Right and Left Ventricle Thrombus in a 68-Year-Old Patient With Sepsis and Cardiomyopathy

**DOI:** 10.7759/cureus.78742

**Published:** 2025-02-08

**Authors:** Batoul Chaaban, Abbas Rachid, Angela N Kanbar, Malek Mohammed, Hasan Kazma

**Affiliations:** 1 Cardiology, Lebanese University Faculty of Medicine, Beirut, LBN; 2 Internal Medicine, Lebanese University Faculty of Medicine, Beirut, LBN; 3 Cardiology, Bahman University Hospital, Beirut, LBN

**Keywords:** blood sepsis, heart failure with reduced ejection fraction, left ventricular cardiac thrombus, right ventricular cardiac thrombus, septic cardiomyopathy

## Abstract

Right ventricular thrombus (RVT) is a rare but clinically significant condition associated with severe complications, such as pulmonary embolism and right heart failure. This case report presents a 68-year-old woman with a history of diabetes, coronary artery disease, and heart failure with reduced ejection fraction (HFrEF), who developed bilateral ventricular thrombi in the context of septic cardiomyopathy secondary to a diabetic foot infection and a urinary tract infection (UTI). Echocardiography revealed thrombi in both the right and left ventricles, severe global hypokinesia, and reduced ejection fraction. The patient was treated with intravenous heparin and showed symptomatic improvement. Diagnostic challenges in RVT were highlighted, emphasizing the limitations of echocardiography in differentiating cardiac masses and the superior diagnostic capabilities of cardiac magnetic resonance imaging (CMR). This case underscores the importance of early diagnosis, individualized management strategies, and the utility of advanced imaging techniques in RVT.

## Introduction

Right ventricle thrombus (RVT) represents a rare yet significant clinical entity that can result in serious cardiovascular issues, such as pulmonary embolism and right heart failure, making prompt diagnosis and treatment critical. The presence of thrombi in the right ventricle is often linked with various underlying conditions, including myocardial infarction, cardiomyopathy, prolonged immobilization, and pulmonary embolism [1,2]. However, the prognosis for patients with right heart thrombus who do not have an associated pulmonary embolism remains ill-defined [2].

Diagnosing RVT poses challenges due to its nonspecific symptoms and the limitations of traditional imaging modalities. Fortunately, recent echocardiography and cardiac MRI advancements have enhanced our ability to detect and manage this condition [3]. This case report will discuss the clinical presentation and diagnostic strategies related to a rare case involving both right and left ventricle thrombus, underscoring the critical importance of early identification and intervention to mitigate potential complications.

## Case presentation

A 68-year-old woman with a medical history of diabetes, coronary artery disease (CAD) status post coronary artery bypass grafting (CABG) in 2018, and heart failure with reduced ejection fraction (HFrEF) on aspirin and Lasix only at home was admitted to the hospital with a diagnosis of sepsis (quick Sepsis Related Organ Failure Assessment=2) secondary to diabetic foot infection with necrosis. Upon arrival at our emergency department, she presented with dyspnea, bilateral lower limb edema, diabetic foot infection, and necrosis, accompanied by symptoms of shortness of breath and a 4 kg weight gain over the past two weeks, likely due to heart failure.

Her vital signs were as follows: respiratory rate of 18 breaths/minute, heart rate of 76 beats/minute, blood pressure of 95/60 mmHg, and oxygen saturation of 95 % on 2 L nasal cannula. Her body temperature was 36.5 °C. The patient was alert and cooperative but showed signs of acute distress. She was oriented to person, place, and time.

**Figure 1:**
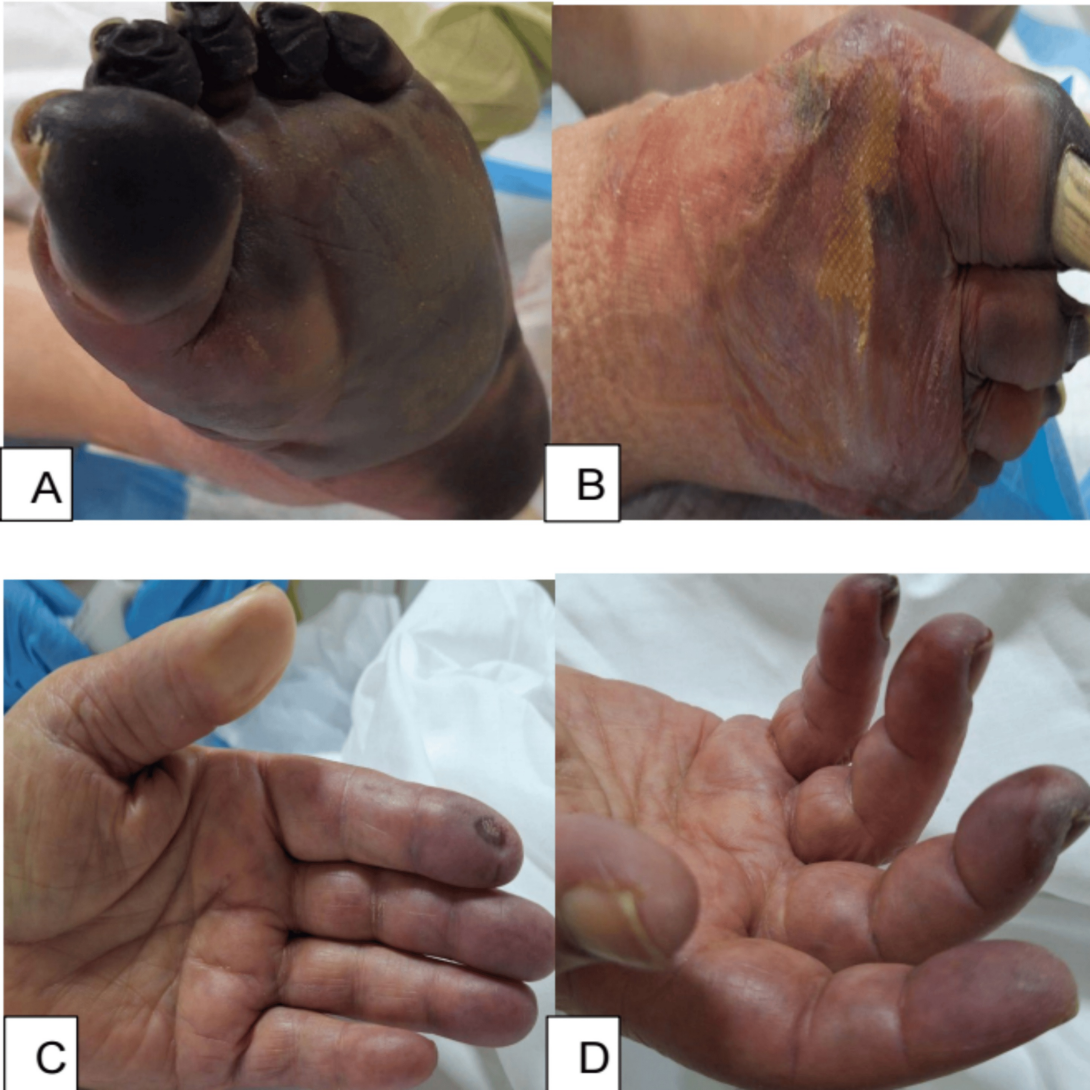
Presentation of upper and lower limbs with ischemia and necrosis A) Left leg; B) Right leg; C) Left hand; D) Right hand

Physical examination of the lungs revealed bilateral rales and diminished breath sounds. Cardiac examination showed normal S1 and S2, and a regular sinus rhythm on the electrocardiogram (ECG); however, there was peripheral cyanosis in her upper and lower limbs, including her toes and fingers. Her extremities were cold and poorly perfused, displaying pronounced edema in the lower limbs and diabetic foot necrosis and infection (Figure 1). Strength and sensation were decreased in limbs, while abdominal examination showed positive bowel sounds; the abdomen was soft, non-distended, and nontender.

Laboratory tests indicated leukocytosis, with a left shift and an increased C-reactive protein level. Serum creatinine was elevated, with the last recorded value in 2018 being significantly lower (2.18 mg/dL), demonstrating a new acute kidney injury. Her serum albumin level was also reduced before presentation. She had completed a 10-day course of broad-spectrum antibiotics for urinary tract infection (UTI) confirmed by urine analysis and culture. The patient exhibited elevated D-dimer levels, possibly due to sepsis. However, thrombotic hypercoagulability should also be considered. A complete hypercoagulability( including antiphospholipid syndrome) workup was not performed due to financial constraints (Table 1).

**Table 1:** Laboratory investigation of our patient during hospitalization SGOT - serum glutamic-oxaloacetic transaminase; SGPT - serum glutamate-pyruvate transaminase; GGT - gamma-glutamyl transferase; PCR - polymerase chain reaction

Parameter	Patient value	Reference range
WBC	12000	4,500-11000/mm3
Hemoglobin	12.3 g/dl	13.5-17.5 g/dl
Platelets	173,000	150-400x10^9^/L
Creatinine	3.07 mg/dl	< 1 mg/dl
BUN	71	< 20 mg/dL
Albumin	2.4 g/dl	4 g/dl
D-Dimer	7.8 ug/ml	0.5 ug/ml
HbA1c	11.2%	6.4%
SGOT	22	45 u/l
SGPT	18	33 u/l
GGT	118	40 u/l
Alk-ph	98	100 u/l
Sodium	132	135-145 meq/l
Potassium	4.2	3.6-5.2 mmoL/L
SARS COVID-19 PCR	Negative	Negative

**Figure 2:**
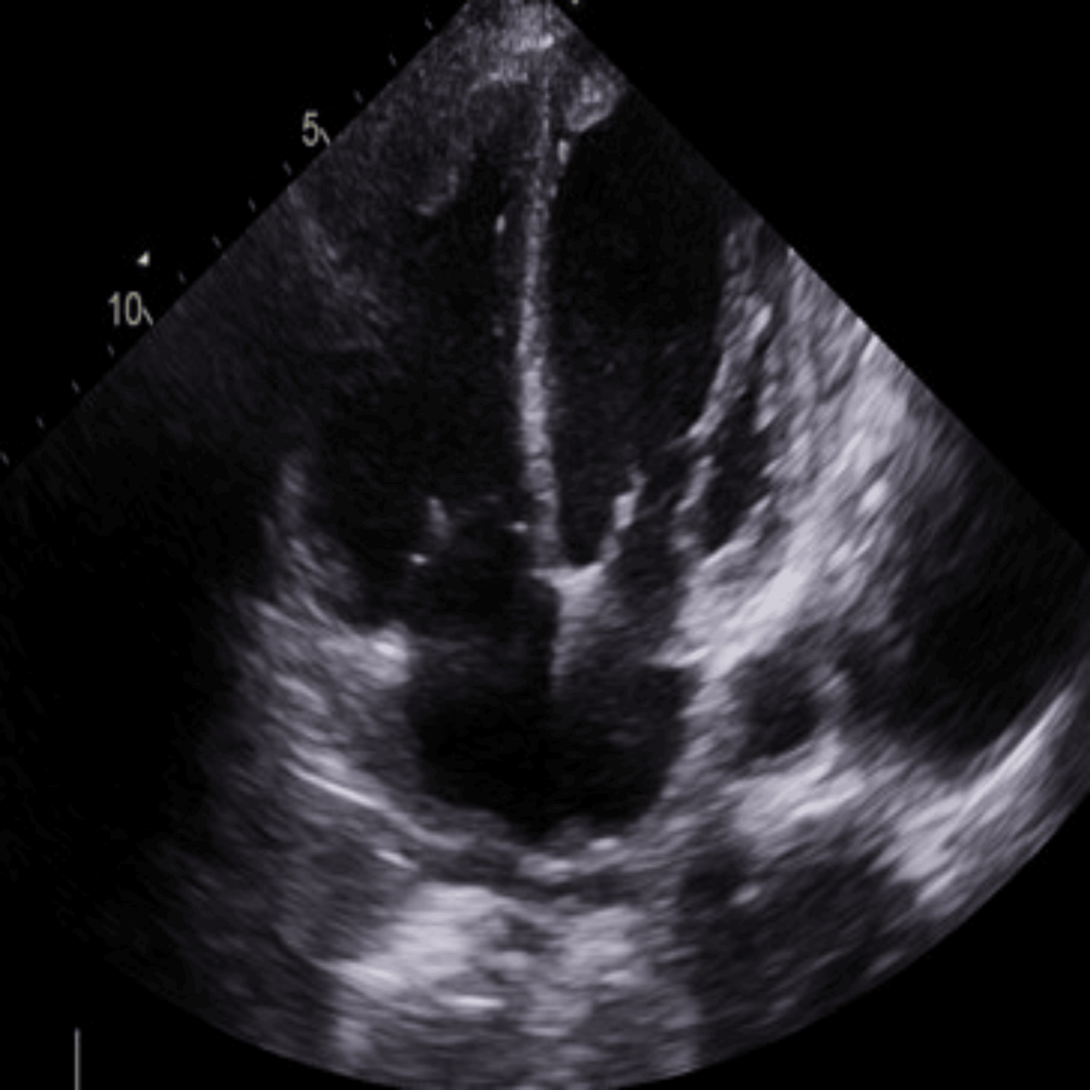
Biventricular thrombi

**Figure 3:**
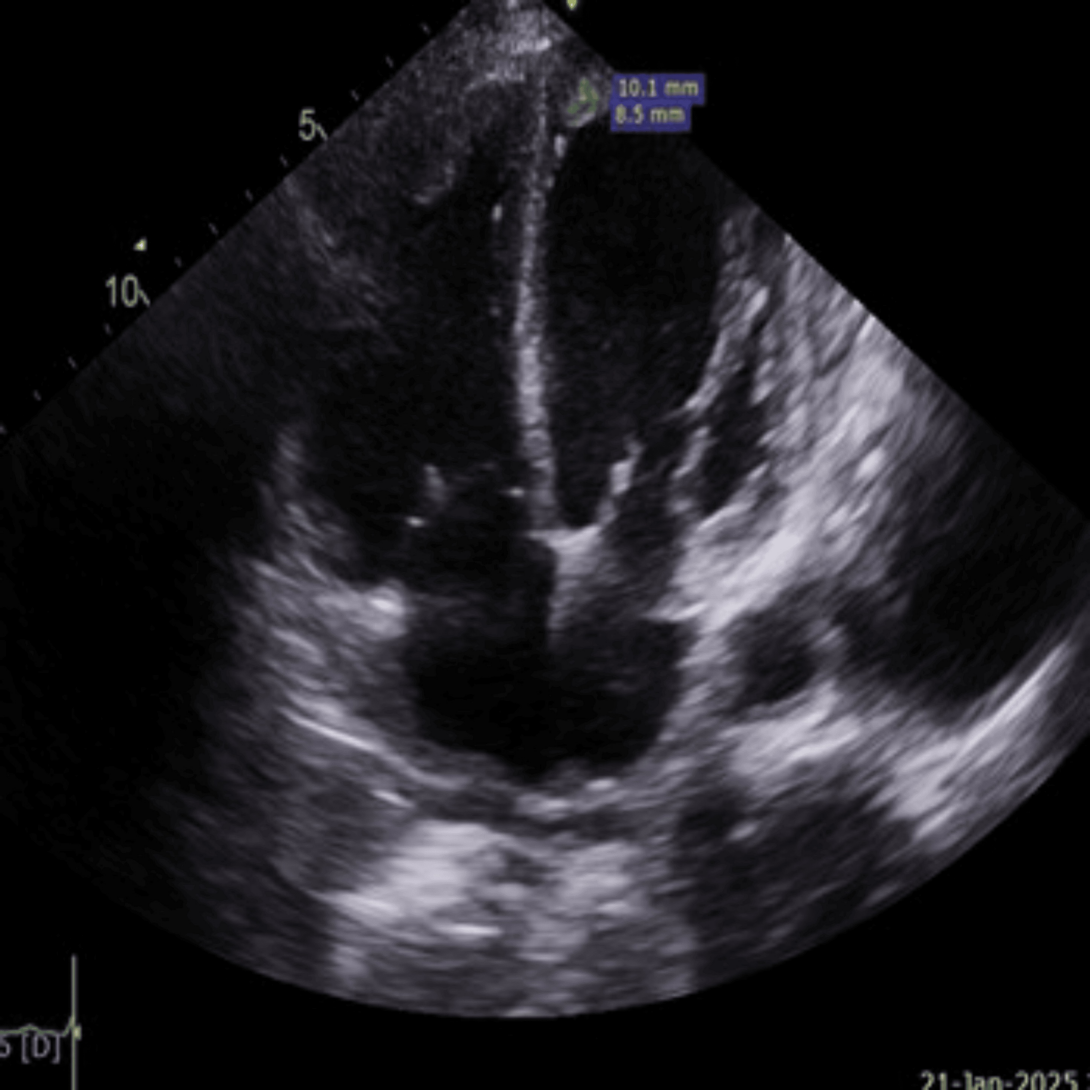
Left ventricular apical thrombus of 10.1 mm x 8.5 mm

**Figure 4:**
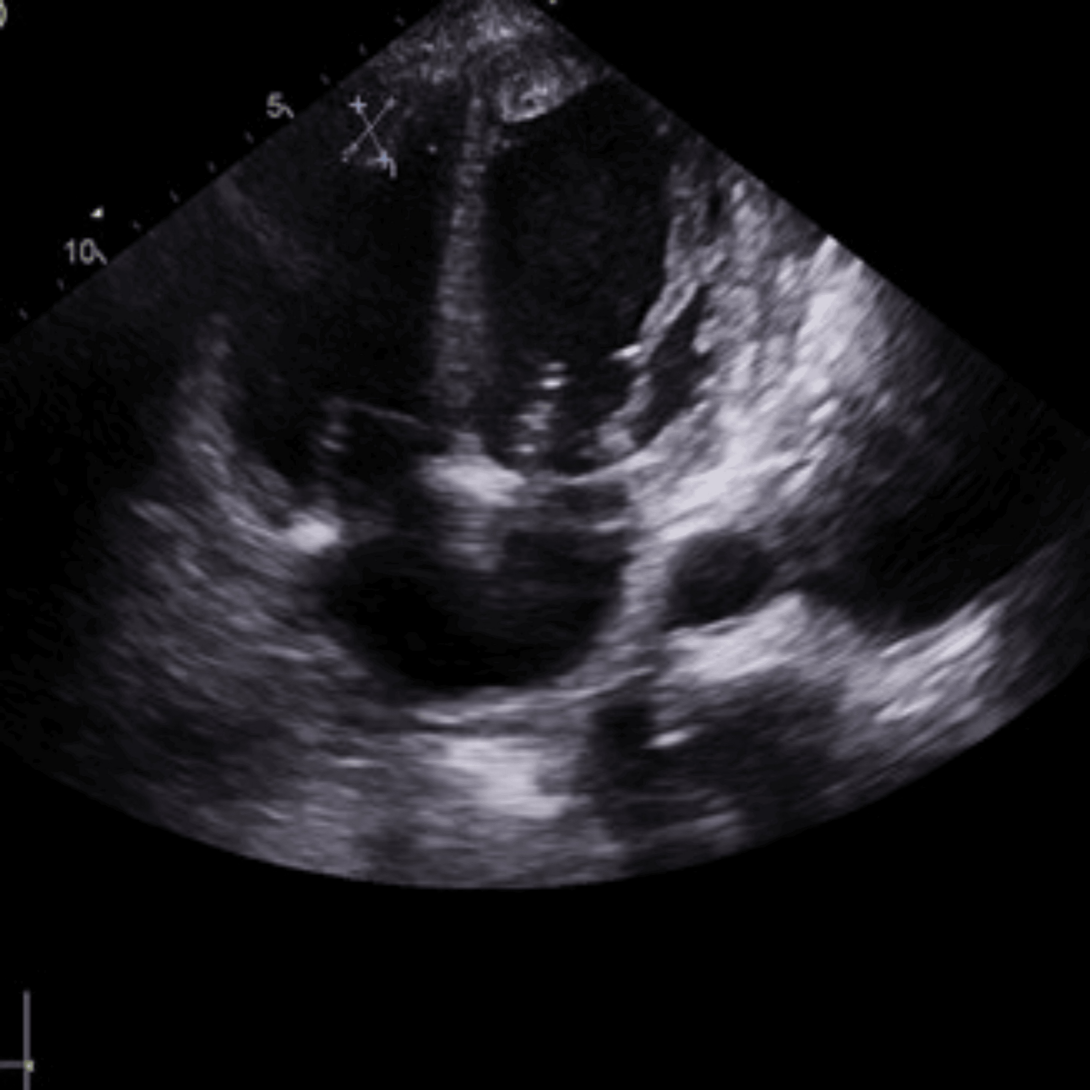
Right ventricular apical thrombus of 18 mm x 11 mm measurement

Further evaluation with echocardiography (TTE) revealed a severely dilated left ventricle (end-diastolic diameter: 5.8 cm, septal thickness: 0.9 cm) with global hypokinesia and an ejection fraction of 15%. A thrombus was observed at the apex of the left ventricle (Figures 2, 3). Similarly, the right atrium and right ventricle were dilated and exhibited reduced function, with another thrombus noted at the apex of the right ventricle (Figures 2, 4). The mitral valve demonstrated grade III mitral regurgitation, while the tricuspid valve showed grade III tricuspid regurgitation with an estimated systolic pulmonary artery pressure of 44 mmHg, likely due to secondary to RV dysfunction. The inferior vena cava (IVC) was dilated, displaying less than 50% collapsibility with inspiration (Videos 1, 2). The aortic valve was calcified without stenosis or regurgitation (velocity: 1.48 m/s), and the ascending aorta appeared normal in size; the pulmonic valve was also normal. Notably, her prior echocardiogram in 2018, post-coronary artery bypass grafting, indicated an estimated ejection fraction of 30-35% with only moderate left ventricular systolic dysfunction, while the sizes of the right atrium, right ventricle, and left atrium were normal.

**Video 1:** Severe global hypokinesia with RV and LV thrombi observed on four-chamber TTE view RV - right ventricular; LV - left ventricular; TTE - transthoracic echocardiogram

**Video 2:** Another four-chamber TTE view demonstrating severe global hypokinesia with biventricular thrombi and reduced EF (15%) TTE - transthoracic echocardiogram; EF - ejection fraction

A chest X-ray showed bilateral congestion suggestive of pulmonary edema accompanied by cardiomegaly and mild right pleural effusion. CT angiography to evaluate for PE was ordered but not performed due to elevated creatinine levels. To avoid worsening renal function during this acute phase, a V/Q scan was also not conducted as it was unavailable at our center; echo Doppler lower extremities arteries and veins was performed at presentation and, after one week, showed no evidence of thrombosis. However, bilateral soft tissue edema in the lower legs was observed. The diagnosis of sepsis and cardiomyopathy was confirmed, though progressive ischemic cardiomyopathy remains a differential diagnosis. The patient was hemodynamically stable with mean arterial pressure (MAP) >65mmhg and was initiated on a treatment regimen consisting of meropenem, Lasix, and intravenous heparin with a target partial thromboplastin time (PTT) of 60-84 seconds.

## Discussion

Right ventricular (RV) thrombus is rare and less well-understood compared to the left ventricular (LV) thrombus, often remaining asymptomatic until complications like pulmonary embolism (PE) or paradoxical stroke develop. In contrast, right atrial (RA) thrombus is about four times more common than RV thrombus and is more frequently associated with medical devices. Patients with RV thrombus, or thrombi involving both the RA and RV, often experience blood stagnation, which is common in individuals with cardiomyopathies or those with foreign bodies, such as catheters and pacemaker leads. Cardiovascular comorbidities, including ischemic heart disease (IHD), heart failure, and chronic kidney disease (CKD), are frequently observed in these patients [4]. Additionally, RV thrombus has been documented in case reports involving COVID-19 and nephrotic syndrome [5,6].

A retrospective study by Goh et al. reported that 55.7% of patients with RV thrombus had indwelling medical devices, such as catheters or device leads [4]. Other predisposing factors associated with RV thrombus include deep vein thrombosis (DVT), atrial fibrillation (AF), cardiomyopathy, and malignancy [4]. In our case, the patient presented with dyspnea both at rest and on exertion, along with pulmonary and lower limb edema, indicating congestion. These symptoms improved with diuretic therapy with a repeated TTE after one week, confirming a decrease in the size of both thrombi. The patient was diagnosed with sepsis and cardiomyopathy using Transthoracic echocardiography (TTE), characterized by severe global hypokinesis, a significantly reduced ejection fraction (EF) of 15%, and the presence of both RV and LV thrombi. Notably, the patient had no catheters or device leads.

Approximately 98% of RV thrombi are associated with PE [7]. In the study of Chartier et al. on a series of 38 patients, PE was confirmed in all but one. DVT was found in 90% of patients who underwent venography or ultrasonography [8]. In contrast, in our patient, the Doppler ultrasound for both limbs showed no DVT, and no arterial stenosis was noted in the examined bilateral femoral and popliteal vein and artery, respectively, with bilateral lower legs diffuse subcutaneous edema.

Diagnosing RV thrombus is challenging, but transthoracic echocardiography (TTE) is often the first imaging technique in an emergency setting; TTE is particularly useful in cases of clinical deterioration, as it may reveal a thrombus that was not detected on the initial examination. If there is any doubt and the diagnostic uncertainty remains, transoesophageal echocardiography (TEE) should be performed, as it is a rapid, safe, semi-invasive bedside procedure. Sometimes, it is necessary to differentiate an RV thrombus from intracardiac tumors, devices, or vegetation. TEE can directly diagnose pulmonary embolism by visualizing a thrombus in the pulmonary arteries and may also identify a patent foramen ovale or even a thrombus trapped within it [8]. While transthoracic (TTE) and transoesophageal echocardiography (TEE) are highly sensitive and specific for detecting left ventricular thrombus, their accuracy is lower for identifying right heart thrombi (RHT), particularly in challenging scenarios such as arrhythmia, obesity, or the posterior positioning of the right ventricle. Additionally, echocardiography cannot reliably distinguish cardiac masses. In contrast, cardiac magnetic resonance (CMR) offers examiner-independent imaging with superior sensitivity and specificity for intracavitary thrombus, utilizing T1/T2-weighted sequences, first-pass perfusion, and gadolinium enhancement to provide detailed tissue characterization. The case series by Barbagallo et al. further underscored the advantages of CMR in detecting RV thrombus, demonstrating its higher sensitivity and specificity compared to standard modalities like TTE and TEE. Barbagallo et al. described three cases where diagnostic uncertainty regarding RV thrombus was resolved using CMR techniques, particularly flow-sensitive phase (FFP) imaging and late gadolinium enhancement (LGE) sequences, which were instrumental in confirming the diagnosis and guiding anticoagulant therapy [3]. In the present case, bilateral thrombi measuring 3 × 3 cm were visualized on TTE. Cardiac magnetic resonance (CMR) was not performed due to its unavailability at our center and the patient's condition, preventing transfer to another facility.

The treatment of RV thrombus remains challenging and varies depending on clinical circumstances. In a study of 177 cases of right ventricular (RV) thromboembolism, Peter S. Rose and colleagues reported an overall mortality rate of 27.1%. The treatment modalities included no therapy (9%), anticoagulation (35%), surgical intervention (35.6%), and thrombolytic therapy (19.8%). Mortality rates differed based on the treatment modality: 100% for no therapy, 28.6% for anticoagulation, 23.8% for surgical embolectomy, and 11.3% for thrombolysis. Notably, thrombolytic therapy was associated with significantly better survival compared to anticoagulation or surgery (p<0.05) [7].

Similarly, a retrospective observational study conducted by Goh et al. found that 84.5% of patients received anticoagulation, while 3.1% underwent surgical thrombectomy; none received thrombolytic therapy. Baseline characteristics were similar between anticoagulated and non-anticoagulated patients. Anticoagulation was associated with significantly lower short- and long-term mortality. Among the non-anticoagulated patients, one developed a paradoxical stroke, while a similar event occurred in one anticoagulated patient. There were no significant differences in the incidence of pulmonary embolism, circulatory collapse, or bleeding complications between the groups. However, anticoagulation status did not appear to influence thrombus resolution [4]. In our case, therapeutic heparin was initiated with a target PTT range of 60-84, with plans to transition to apixaban according to her glomerular filtration rate upon discharge.

## Conclusions

This case highlights the clinical and diagnostic challenges associated with right ventricular thrombus, a rare but life-threatening condition. Early recognition of RVT through imaging, particularly with advanced modalities such as cardiac magnetic resonance imaging, is critical for accurate diagnosis and effective management. The treatment of RVT remains complex and requires an individualized approach based on patient characteristics and clinical context. In this patient, prompt initiation of therapeutic anticoagulation with heparin resulted in symptomatic improvement. It is crucial to consider right ventricular thrombosis in septic cardiomyopathy, as not all RV thrombi are linked to deep vein thrombosis, as demonstrated in this case. This case underscores the importance of vigilance in high-risk patients and the integration of advanced diagnostic tools to guide therapeutic decisions and improve outcomes.

## References

[1] Leow AS, Sia CH, Tan BY, Chan MY, Loh JP (2020). Characterisation of patients with acute myocardial infarction complicated by left ventricular thrombus. Eur J Intern Med.

[2] Watson NW, Weinberg I, Dicks AB (2023). Clinical significance of right heart thrombus with and without an associated pulmonary embolism. Am J Med.

[3] Barbagallo M, Naef D, Köpfli P (2021). Right ventricular thrombus, a challenge in imaging diagnostics: a case series. Eur Heart J Case Rep.

[4] Goh FQ, Leow AS, Ho JS (2022). Clinical characteristics, treatment and long-term outcomes of patients with right-sided cardiac thrombus. Hellenic J Cardiol.

[5] Mitsis A, Alexi A, Constantinides T, Chatzantonis G, Avraamides P (2022). A case of right ventricular thrombus in a patient with recent COVID-19 infection. Cureus.

[6] Lempp S, Schwenger V (2017). Isolated right ventricular thrombus in an adult patient with nephrotic syndrome: a case report. J Med Case Rep.

[7] Rose PS, Punjabi NM, Pearse DB (2002). Treatment of right heart thromboemboli. Chest.

[8] Chartier L, Béra J, Delomez M (1999). Free-floating thrombi in the right heart: diagnosis, management, and prognostic indexes in 38 consecutive patients. Circulation.

